# Supplementary material for: Novel Driver Strength Index highlights important cancer genes in TCGA PanCanAtlas patients
Source: PeerJ. 2022 Aug 11;10:e13860. doi: 10.7717/peerj.13860 (PMC9375969; doi:10.7717/peerj.13860)
Supplement: Supplemental Information 2 [file peerj-10-13860-s002.zip › COHORTS/genes plots/cohorts/2021_8_16_14_9_distribution_cohorts_females_HNSC.pdf]

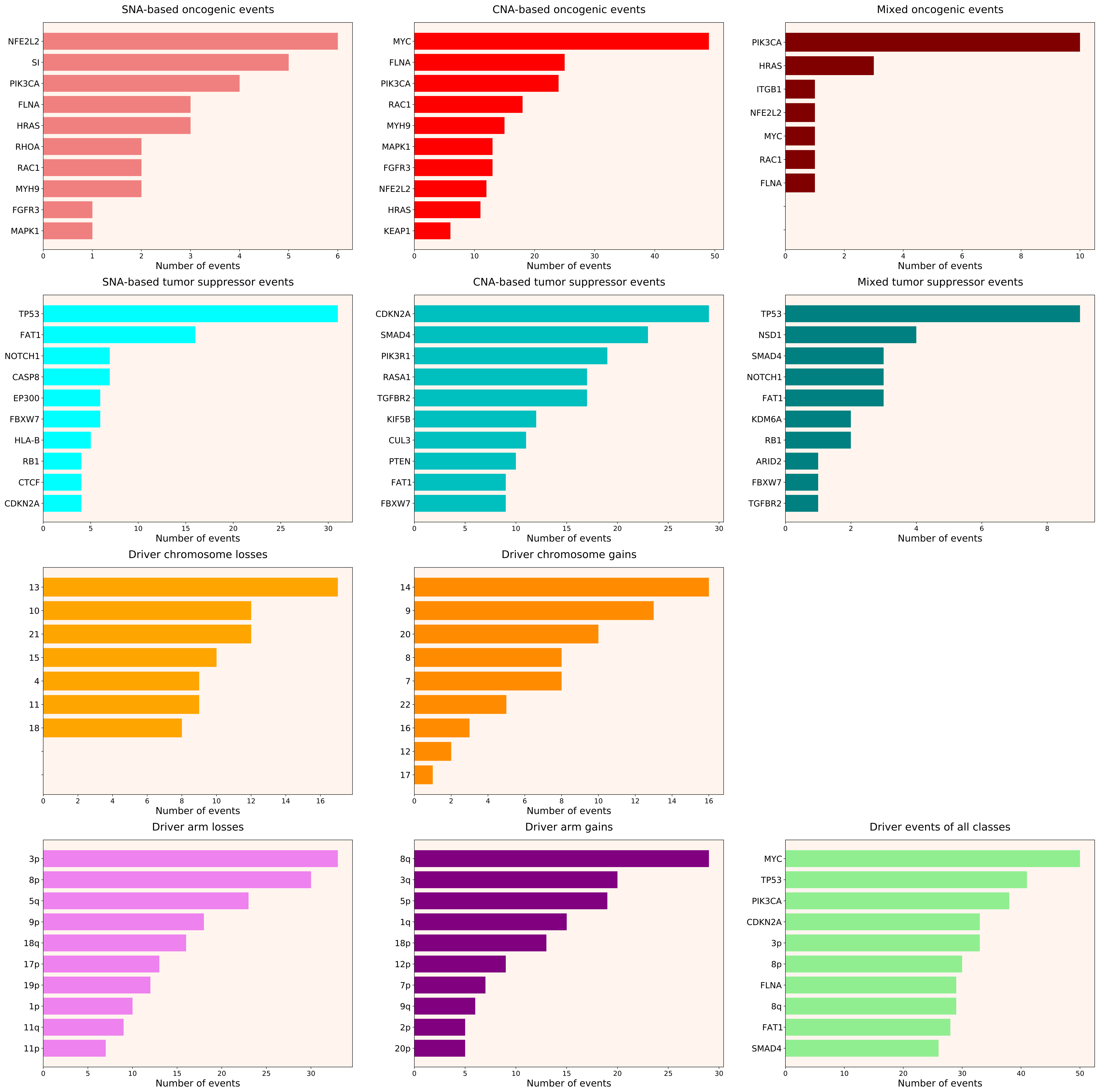
SNA-based oncogenic events
NFE2L2
SI
PIK3CA
FLNA
HRAS
RHOA
RAC1
MYH9
FGFR3
MAPK1
0 1 2 3 4 5 6
Number of events
CNA-based oncogenic events
MYC
FLNA
PIK3CA
RAC1
MYH9
MAPK1
FGFR3
NFE2L2
HRAS
KEAP1
0 10 20 30 40 50
Number of events
Mixed oncogenic events
PIK3CA
HRAS
ITGB1
NFE2L2
MYC
RAC1
FLNA
0 2 4 6 8 10
Number of events
SNA-based tumor suppressor events
TP53
FAT1
NOTCH1
CASP8
EP300
FBXW7
HLA-B
RB1
CTCF
CDKN2A
0 5 10 15 20 25 30
Number of events
CNA-based tumor suppressor events
CDKN2A
SMAD4
PIK3R1
RASA1
TGFBR2
KIF5B
CUL3
PTEN
FAT1
FBXW7
0 5 10 15 20 25 30
Number of events
Mixed tumor suppressor events
TP53
NSD1
SMAD4
NOTCH1
FAT1
KDM6A
RB1
ARID2
FBXW7
TGFBR2
0 2 4 6 8
Number of events
Driver chromosome losses
13
10
21
15
4
11
18
0 2 4 6 8 10 12 14 16
Number of events
Driver chromosome gains
14
9
20
8
7
22
16
12
17
0 2 4 6 8 10 12 14 16
Number of events
Driver arm losses
3p
8p
5q
9p
18q
17p
19p
1p
11q
11p
0 5 10 15 20 25 30
Number of events
Driver arm gains
8q
3q
5p
1q
18p
12p
7p
9q
2p
20p
0 5 10 15 20 25 30
Number of events
Driver events of all classes
MYC
TP53
PIK3CA
CDKN2A
3p
8p
FLNA
8q
FAT1
SMAD4
0 10 20 30 40 50
Number of events
